# Pleistocene Hypothesis – Moving Savanna Perceptual Preference Hypothesis Beyond Savanna

**DOI:** 10.3389/fpsyg.2022.901799

**Published:** 2022-05-30

**Authors:** Joachim Rathmann, Kalevi M. Korpela, Philipp Stojakowits

**Affiliations:** ^1^Institute of Geography, Augsburg University, Augsburg, Germany; ^2^Faculty of Social Sciences/Psychology, Tampere University, Tampere, Finland

**Keywords:** savanna hypothesis, landscape preferences, human health-environment relationships, paleoanthropology, therapeutic landscapes

## Abstract

We provide an extension of the Savanna perceptual preference hypothesis (“Savanna Hypothesis”), supposing that interaction with landscapes offering survival advantage for human groups during evolution might have gradually evolved to permanent landscape preferences. This additional support is based on the palaeoenvironmental analysis of the spread of modern humans into Europe in the late Pleistocene and their living environments there. Our hypothesis is that the preference for park-like landscapes after African savannas experienced a kind of “refreshment” in the Pleistocene. Thus, preferences for certain types of natural settings and scenes may have a more continuous evolutionary history than previously thought. The extended Savanna Hypothesis termed “Pleistocene Hypothesis” might stimulate further work on this important topic linking human evolution and human environmental preferences.

## Introduction

In this paper, we provide an extension of the perceptual Savanna Hypothesis supposing that human interaction with landscapes offering survival advantage during evolution might have gradually developed into enduring patterns to prefer certain landscapes. This additional support is based on the palaeoenvironmental analysis of the spread of modern humans into Europe and their living environments there. Different factors play a role in this process (Klein, 2008). The role of climate forcing is one of them (e.g., Müller et al., 2011; Staubwasser et al., 2018). Another aspect is the innovation dynamic (Shennan, 2001; Richerson et al., 2009), i.e., the acquired unique abilities to colonize new environments (Hoffecker, 2009). The spread of early hominids can, however, also be explained by the acquisition of food, being essential for the development of the brain as the dispersal of early hominids is correlated to the historical migration of the African buffalo (van Ginneken et al., 2017; van Ginneken, 2019). In this context, theories on the evolution of the human brain should further discuss the current way of landscape perception in relation to that of the hominids (Chin et al., 2022).

But finally, when modern humans arrived in a new area what preferences did they have to answer the question: should I stay or should I go (further)?

The savanna hypothesis was originally formulated as an answer to why humans are bipedal. Thus, it places the agents of selection for bipedality on open grasslands, resulting from the transition of the human ancestors from an arboreal lifestyle to one on the savannas in response to the opening of the African landscape during long-term aridification of the continent (Richmond et al., 2001). The second aspect of this hypothesis focuses on the psychological processes assuming that due to the long-term hominization in the East African savanna, such environments are part of general landscape preferences of humans. The basic claim of the Savanna Hypothesis is that humans have innate, even automatic tendencies toward preferring certain types of natural settings that provide food, water, and security for survival (Ulrich, 1993). Ulrich (1983) has presented an integrated theory of esthetic and affective response to the natural environment assuming that landscape preferences can be defined as “the first level of reaction to the environment of generalized affect, such as liking or interest” simultaneous with or followed by approach-avoidance behavior (Ulrich, 1983, p. 90). These affective reactions to natural scenes are adaptive and foster well-being in terms of the total behavior of the individual. The initial affective reaction is elicited quickly by certain general properties or preferenda of the view, including gross structural aspects of settings, (e.g., focality, deflected vistas), gross depth properties that require little inference (spaciousness, ground surface texture conducive to movement) and general classes of environmental content, such as vegetation and water. Thus, in this theory, preferences are linked *via* preferenda to survival and well-being. Evolutionary psychology argues that many psychological mechanisms, such as preferences and emotions and behavioral strategies, are solutions to the adaptive problems our species has faced in natural evolution (Buss, 1995). We believe that the scientific basis of a widespread Savanna hypothesis requires further study and we focus on theoretical research of evolutionary biology theories.

## Fear and Preference, Savanna Hypothesis and Prospect Refuge Theory

For the longest time in human history our ancestors lived in hunter–gatherer bands, thus being intimately connected to other living organisms (Wilson, 1984). Automatic reactions of fear and escape after the detection of predators were important agents of selection on ancestral humans (Öhman and Mineka, 2001). Many studies stress the evolutionary origins of human fear of some animals and show evidence that people detect the presence of fear-relevant animals (i.e., snake, spider) faster than the presence of a pleasant stimulus (i.e., flower, mushroom) (Öhman et al., 2001; Lipp et al., 2004). Even preschool children share this special attention to snakes and detect them faster than other stimuli (i.e., flowers, frogs, and caterpillars) (LoBue and DeLoache, 2008). Thus, it forms an evolutionary advantage to recognize dangers quickly and to react to them as efficiently as possible. Therefore, wilderness may also be partly associated with death and mortality (Koole and Van den Berg, 2005). On the other hand, research literature on preferences for natural settings shows that people generally prefer landscapes with a variety of landscape structures, open green spaces, differences in relief that enable views and orientation, as well as loose trees and the presence of water (Ulrich, 1993; Hill and Daniel, 2007).

Such preferences seem to relate not only to savanna but rather to mosaic environments (Domínguez-Rodrigo, 2014) and we develop this line of argument by first looking at the evidence on the preference of natural vs urban landscapes and then proceed to the preferred types and features of natural environments.

Several studies have consistently found empirical support for the assumption that people respond more positively to natural vs. urban environments (Staats et al., 2003; Van den Berg et al., 2003; Berto, 2005). Urban environments in these studies refer to human-made, built environments, such as streetscapes and buildings. Many links between human well-being and nature contacts have been analyzed and numerous studies have highlighted the positive effects of different kinds of green spaces on human health, well-being and quality of life (Hansmann et al., 2007; Abraham et al., 2010; Hartig et al., 2014; McMahan and Estes, 2015; Ideno et al., 2017; Twohig-Bennett and Jones, 2018; Bell et al., 2018; Grilli and Sacchelli, 2020; Rathmann et al., 2020; Rathmann, 2021). Supporting the notion of potentially hard-wired differences in brain activity, it has been shown that lower attentional demands for natural versus urban images can be detected within 1,200 milliseconds (Grassini et al., 2019; see also Norwood et al., 2019). Rapid positive emotional reactions to natural scenes versus negative reactions to urban scenes are evoked in 200 ms (Korpela et al., 2002). Savanna preference hypothesis also assumes that along with the preference reaction during evolution, a capacity for restorative, i.e., stress-reducing responding to certain natural settings has developed (Ulrich, 1993). This would foster amelioration of stress responses after encounters with danger and threats, such as predators. Ample evidence shows that stress-reducing physiological responses, indexed by, e.g., heart rate variability, salivary cortisol, blood pressure and pulse rate, are detected more rapidly after a stressful situation while walking or sitting in natural settings than in urban settings (Park et al., 2010; Bratman et al., 2012; Ideno et al., 2017; Twohig-Bennett and Jones, 2018; Mygind et al., 2019). The perception of environments’ “naturalness” and its biodiversity has also been associated with the restorative qualities of a setting (Carrus et al., 2015).

To continue to the preferred types and features of landscapes, the theory of prospect and refuge can be traced back to the late nineteenth century and the anthropological belief in the human survival instinct leading to a stimulus, which directly connects human perceptions or reactions to environmental stimuli. The basic idea, that hominids have been living in the Savanna environments has started to be discussed since 1960, at least in archeology and history (Bender et al., 2012), underlining the paleoanthropological perspective of the Savanna theory. The psychological perspective of this theory focuses on the needs of the hominids for survival.

The prospect-refuge theory, proposed by Appleton (1975, 1996), describes a human behavioral and psychological need for places that allow a person to see, but without being seen. The basic idea was to show a simple model, relating preferences to a typology of landscapes based on behavioral and biological sciences. The whole model was “an agent of simplification for explanatory purposes” (Appleton, 1984, p. 92). The reduction was made deliberately to facilitate an explanation. Environmental perception is the key to all adaptation processes and humans perceive their environment in another way than animals perceive their habitat. Some aspects of the habitat are more important for survival than others. “Prospect” and “refuge” are the important aspects of a landscape to improve the chances of survival. Further on, this theory tried to show a biological interpretation for landscape esthetics as it states that taste in art is an acquired preference for particular methods of satisfying inborn desires which are basically opportunity (prospect) and safety (refuge). Humans are attracted to specific circumstances (art, landscape) that have unoccluded vistas into the landscape (prospects further include hills, mountains and trees), visible places for easy refuge (e.g., climbable trees with dense canopies nearby, caves, dense vegetation) and additionally water, plants, prey species. Landscape preferences further include spaces, where we are rather on the edge than in the middle of a place, where we are most exposed and places where we are covered, compared to the open sky. Landscape preferences therefore focus on areas which are optimal for survival and reproduction. The Savanna Hypothesis (Orians, 1980; Bender et al., 2012) argues that selection favored resource-rich environments whereas environments, lacking resources or with survival threats have been avoided. Such environments offered the essential landscape characteristics for survival of the early humans; the availability of resources, protection against predators, the possibility of orientation and overview in space are central requirements for a landscape that ensures the survival of early humans. These theoretical conceptions actually propose that not only certain landscape contents but certain structural properties might have been important for the development of permanent preferences. One such property has been described as “depth/spaciousness” characteristics that relate to surveillance, proximity to hidden threats, and escape opportunities (Ulrich, 1993) or intermediate complexity or density of the scene (Joye and van den Berg, 2011). Such structural emphasis may also fit with alternative theoretical explanations for the Savanna Hypothesis, such as the Perceptual Fluency Account (PFA). It states that natural scenes are affectively evaluated more positively than urban scenes because our visual system more fluently processes certain aspects of the visual *structure* of the former than of the latter (Joye and van den Berg, 2011). In PFA, in contrast to psychophysiological and emotional theories supporting Savanna Hypothesis, it is the structure of the landscape, visual coherence and fractal patterns, rather than “unthreatening vegetated settings *per se*” that might explain preference of greenspace (Joye and van den Berg, 2011).

Emphasizing contents rather than structure, Heerwagen and Orians (1993) argue that our landscape preferences are innate, these preferences include open spaces of low grasses, the presence of water, flowering and fruiting plants, and evidence of animal life. Beside the prospect-refuge-theory and the Savanna Hypothesis, Kaplan’s landscape preference matrix theory (Kaplan and Kaplan, 1989) and the stress recovery theory (Ulrich et al., 1991) argue that humans prefer natural green environments. More importantly and in line with the concept of mosaic environments, in addition to the Savanna hypothesis, two other hypotheses with respect to the specific habitat where humans have evolved have been presented (Han, 2007; Mangone et al., 2021). One option is the forest hypothesis, which argues that human evolution took place in closed, forested settings and the other one is the grassland–woodland hypothesis, which proposes that a mosaic of both settings was the adaptive environment for hominids.

Some of the empirical evidence for the preference for savanna environments is related to biomes as a whole but mainly it includes only some features of savanna environments. Concerning biomes, the evidence is mixed. In one study, photographs of five biomes, rain forest, deciduous forest, coniferous forest, savanna, and desert were rated by the inhabitants of the rainforest belt of Nigeria (Falk and Balling, 2010). The results showed that savanna scenes were regarded as the most favorable place to live. In another study, college students’ psycho-physiological responses to the six major terrestrial biomes (desert, tundra, grassland, coniferous forest, deciduous forest, and tropical forest) showed that tundra and coniferous forest were the most favored biomes, whereas desert and grassland were the least favored (Han, 2007). Yet another study with a student population showed that irrespective of familiarity, beaches and lakes were preferred more and marshes and swamps preferred less than the other six biome types (beach, lake, tropical and temperate forest, marsh, swamp, meadow, park as a representative of savanna, mountain, and river) (Mangone et al., 2021). Concerning features of savanna, there is evidence on cross-cultural preferences for acacia-like Savanna trees (Orians and Heerwagen, 1992). Lohr and Pearson-Mims (2006) consider people’s preference for trees with spreading cones comparable to an acacia. Sommer (1997) confirms this result within a cross-national study thus confirming the refuge dimension of Appleton’s theory as such a canopy can represent habitat and safety. Anecdotal evidence points to the fact that looking at trees outside a hospital room helps in recovering more quickly from hospital stay than patients looking at a brick wall (Ulrich, 1984).

Moreover, the general question of the degree of our landscape preferences and behavior being innate versus learned during childhood is a part of a long debate. Humans have adapted to a broad range of conditions as social, cultural and natural selection unfold in tandem (Hartig, 2021). Thus, landscape preferences might be determined by culture as some studies stress a relationship between childhood memories and preferences for specific environments (Van den Berg et al., 1998) and others emphasize cultural determinants (Bourassa, 1992; Howley, 2011; Joye and van den Berg, 2011; Ward Thompson, 2018; Silva et al., 2020; Hartig, 2021). Landscape preferences, explained by cultural-based elements must first explain the concept of landscape, and these discussions have a very long tradition, especially in geography (Cosgrove, 2004; Wylie, 2007). The evolution of landscapes must be regarded as a reciprocal interplay of both ecological and cultural “factors.” Carl Sauer’s much-cited work on Sauer (1925) stresses the active agency of culture in shaping landscapes, rejecting environ mental determinism. Further, landscape is the esthetically perceived environment, therefore it affects human wellbeing. Wellbeing is regarded as an important factor in pro-environmental behavior across different cultures and countries (Capstick et al., 2022) stressing the importance of human-nature bonds for environmental policy.

Due to the complexity of the concept of landscape, it must be distinguished from concepts such as place, space, or territory in a political context (Menatti and Casado da Rocha, 2016). Landscape is the existentially experienced environment as a result of history, reflecting economy and society. And so are landscape preferences in this perspective.

The discussions about the different concepts of landscape are far beyond the scope of this contribution but such concepts of landscape offer the possibility of reconnection ecology, and psychology to current humanities concerns with culture, identity, meaning, and even ethics (Menatti and Casado da Rocha, 2016). Environmental psychology is increasingly recognizing that human–environment interactions are culture-bound (Tam and Milfont, 2020), thus discussing the cultural factors in landscape perception, preferences, and individual wellbeing.

Hartmann and Apaolaza-Ibáñez (2013) confirm the role of familiarity as there are preferences for images of lush green landscapes with water and familiar biomes (see also Mangone et al., 2021). Human nature attachment has been related to both evolution and cultural bonds to places, as landscape preferences are related to places where human beings feel safe and at home (Adevi and Grahn, 2012). Differences in preferences for nature between demographic groups appear to be small (Stamps, 1999) but differences in landscape preferences between Western students and non-Western students have been found (Hägerhäll et al., 2018) or preferences for tropical forest landscapes (Moura et al., 2018). Also, age-related changes in landscape preferences have been found (Balling and Falk, 1982). Following from this “innate-learned debate” and the complex mix of social, cultural, and natural selection, our focus in this paper on a special preference for certain types of natural settings does not exclude or oppose potential preferences for (or evolutionary adaptation to) urban settings; neither do we maintain that preferences (= visual inclinations to prefer) for nature *per se* necessarily signify particular health benefits from nature (cf. Hartig, 2021). Although there is ample evidence that such health or wellbeing benefits do exist particularly in comparison to urban scenes (Abraham et al., 2010; Hartig et al., 2014; Twohig-Bennett and Jones, 2018; Mygind et al., 2019) we emphasize here Ulrich’s (1983) theoretical notion that an individual’s affective reaction to a natural scene serves only as an action impulse for adaptive behavior which can be suppressed or denied, based on experience and learning.

To conclude, empirical evidence for preference for savanna-like environments is only partial and several types and features of natural environments attracting human preference have been presented. Thus, a very important and interesting observation in this respect is Domínguez-Rodrigo’s (2014) notion, based on palaeoecological evidence, that Savannas should be regarded as mosaic environments and not as open grasslands.

Several studies also show that human evolution took place in different biomes and not only in savanna environments. Hominids evolved in East Africa in an ecologically diverse setting, including grassland, savanna and different forest structures (Kingston et al., 1994). But whereas the early Miocene mammalian faunas had a tropical-forest character, the Pliocene shows more faunas evolving a savanna-mosaic character (Cerling et al., 1997). But as the interactions between climate change, ecology, and evolution are rather complex, more studies are needed to investigate this complex interplay (Blumenthal et al., 2017).

We will further explore the possibilities of extending the Savanna Hypothesis including various environments with various structural properties, such as prospect and refuge, from an evolutionary perspective. With all the uncertainty in the reconstruction of the palaeoenvironmental conditions during hominization, the Savanna Hypothesis could be given new perspective by investigating the Pleistocene.

## Beyond Savanna

**FIGURE 1:**
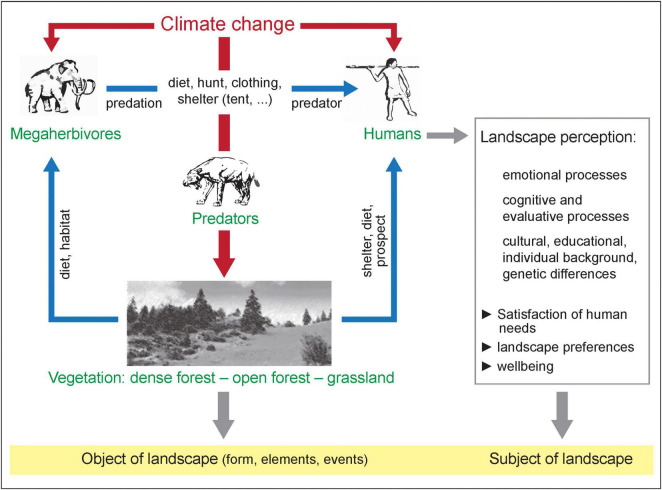
The simplified depiction of the interactions of mankind, climate change, the impact of mega herbivores on the landscape structures and the subjective perception of landscape. The focus is on the interactions between climate and the biosphere with human forcings affecting the environment and actively shaping their environment. Source: Winfried Weber, Institute of Geography and Geology, University of Würzburg, 2022. Reproduced with permission.

In the warm periods of the Pleistocene, many of the northern hemispheric forest areas were apparently open woodlands with large open spaces, structurally somehow similar to Savannas. Based on the mega herbivore hypothesis, the forests were then under high grazing pressure from mega herbivores, i.e., large herbivores (e.g., forest elephants, forest rhinos, giant deer), which, depending on the author, are defined as weighing more than 800, 900 or 1,000 kg (Malhi et al., 2016; van Valkenburgh et al., 2016). During the Pleistocene cold periods, however, largely open landscapes with other mega herbivores (e.g., wooly mammoths, wooly rhinoceroses, musk oxen, giant sloths) dominated. Generally, an alternation between open steppe/tundra and forested landscapes took place affecting human evolution (Sanchez Goñi, 2020). Within a glacial period, warmer phases occurred in which not only tundra or steppe biomes were present, but also some patches of woodland and shrub stands leading to a complex mosaic of different plant communities. The best modern analog for this Pleistocene steppe-tundra environment is located in the Altai Mountains (Chytrý et al., 2019). Figure 1 illustrates the complex interactions of mankind, climate change, the impact of megaherbivores on the landscape structures and the subjective perception of landscape.

**FIGURE 2:**
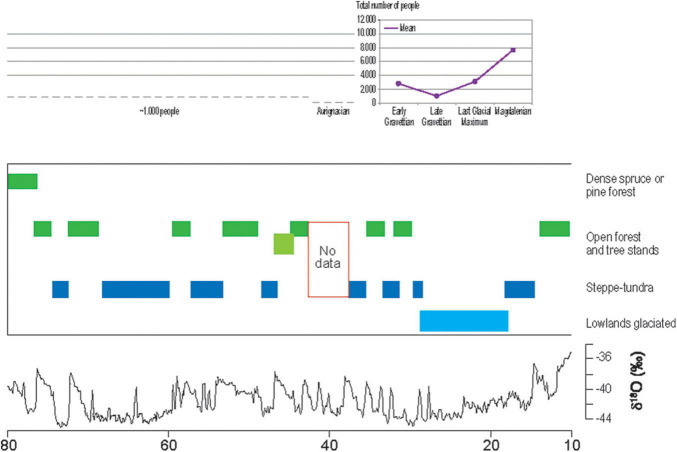
Vegetation and palaeoclimatic development in comparison to the estimated population from c. 80 to 10 ka (x axis). According to Heiri et al. (2014), with additional palaeobotanical data (Müller et al., 2003) and estimated total number of people for the Upper Danube region until c. 42 ka (Müller-Beck, 1983), for the same region during the Aurignacian until c. 33 ka (Schmidt and Zimmermann, 2019), the early Gravettian until c. 29 ka and late Gravettian until c. 25 ka (Maier and Zimmermann, 2017), the LGM (Maier et al., 2016), and Magdalenian (Kretschmer, 2015; Maier, 2017). The Greenland ice core oxygen isotope record NGRIP is shown in the lower part of the figure according to Svensson et al. (2008), supplemented by Rasmussen et al. (2014).

At the time of the last cold period, the early modern human settlement of southern Central Europe occurred during Marine Isotope Stage 3 in a medium-cold steppe-like environment with some boreal trees at climatically favorable sites (Nigst et al., 2014). The rare palynological studies of Southern Central Europe confirm this vegetation reconstruction (Brande, 1982; Müller et al., 2003; Stojakowits et al., 2021; Figure 2). Bielinis et al. (2018, 2019)—based on the salutogenic landscape preference framework—have shown that even snow-covered forests and broad–leafed forests in winter can trigger positive emotions and can lead to psychological relaxation. Another study suggests lowering of blood pressure and even immune system effects of forests in winter (Peterfalvi et al., 2021). Thus, we propose that Late Pleistocene environments might have partly contributed to the development of landscape preferences, although the most ancient preferences of *Homo sapiens* were formed in Africa during the two last glaciations (Riss and Würm) and interglacial stages (Mindel-Riss and Riss/Würm). The first traces of modern humans date to around 315,000 years ago (Richter et al., 2017).

According to genetic studies, modern humans likely mixed with Neanderthals (Prüfer et al., 2014). Both, the early modern humans and the Neanderthals were shaped by open landscapes over many generations. After the ice retreat Mesolithic tribes lived in open landscapes until the reforestation with *Betula* trees took place in the Bølling Interstadial at around 14.5 ka (Eusterhues et al., 2002). Later on, *Pinus sylvestris* also reimmigrated forming open pine-birch-forests until the onset of the Boreal at around 10.3 ka. Around 5,500 cal BC, the colonization of Neolithic tribes of the Linearbandkeramik (LBK) along the Danube started in Southern Central Europe (Gronenborn, 2004). These people used to live in open grass steppe landscapes as testified by pollen analyses (e.g., Wick et al., 2003; Litt et al., 2009). They created similar kinds of open landscapes in their newly settled areas on a small scale- which were densely forested at that time—in order to practice farming. Open lands are preferred settlement regions for humans over many thousands of years dating back to the Late Pleistocene. Although wilderness seems to be more fascinating than cultural landscapes (Barbiero and Berto, 2021) domestication of animals and plants during the Neolithic period might have contributed to favoring the cultural landscapes, over the wilderness landscapes.

## Conclusion

Although the Savanna Hypothesis can be questioned in many ways, it may be extended by the integration of Pleistocene environments. Our hypothesis is, that the preference for mosaic, park-like landscapes experienced a kind of “refreshment” in the Pleistocene. We suggest a heuristic tool, as Appleton did, and not a comprehensive explanation of human behavior and perception, being aware that landscape preferences are not simply based on innate response to the environment. The extended Savanna Hypothesis, termed “Beyond-Savanna-Hypothesis” or according to our study “Pleistocene-Mosaic-Environments-Hypothesis,” might also stimulate work linking human evolution, human health, and general human-environment bond (c.f., Mangone et al., 2021; Chang et al., 2022). We propose empirical studies that would account for both genetic, environmental and developmental influences and their complex interactions on such preferences.

## Data Availability Statement

The original contributions presented in the study are included in the article/supplementary material, further inquiries can be directed to the corresponding author/s.

## Author Contributions

JR: basic idea, literature review, and text. KK: text on psychology. PS: text on paleoenvironments. All authors contributed to the article and approved the submitted version.

## Conflict of Interest

The authors declare that the research was conducted in the absence of any commercial or financial relationships that could be construed as a potential conflict of interest.

## Publisher’s Note

All claims expressed in this article are solely those of the authors and do not necessarily represent those of their affiliated organizations, or those of the publisher, the editors and the reviewers. Any product that may be evaluated in this article, or claim that may be made by its manufacturer, is not guaranteed or endorsed by the publisher.
